# Address to United States Veterinary Medical Association

**Published:** 1889-10

**Authors:** Rush S. Huidekoper

**Affiliations:** Vet., Retiring President


### Art. XXI.—ADDRESS TO UNITED STATES VETERINARY MEDICAL ASSOCIATION.

By Rush S. Huidekoper, Vet.,
*Retiring President.*

Gentlemen :—On reaching the end of another year of the life of this Association, during which I have had the honor to occupy your President's chair, I found so little accomplished by myself or by the Association as a body, that I at first hesitated about referring to it at all, but, upon looking back over the growth of the Association, I came to the conclusion that I should not be too meek, for perhaps, I could find something which would justify me in having some pride and sharing it with my colleagues.

At least the Association was larger than it had ever been before ; it was more travelled, its meetings had been held outside of New York and Boston ; if, as a body, it had not advanced anything to startle the world, at least some of its members had brought themselves usefully and prominently before the public, and we could appropriate some of their reflected glory, and shine ourselves. By this time I was luminous with the achievements and dignity of the Society, which I had the honor to represent. I was a Pharisee on a pinnacle, and, imbued with vain-glorious ardor, undertook to formulate our acts into words that would be grateful to you and satisfying to myself. I knew what certain members of our body had been doing of credit to themselves and their communities, but where was the marked advance that would separate the last from all other years, the brilliant discovery that would mark one of the cycles of the history of Veterinary Medicine, the famous book that would adorn all medical libraries in the future and be a famous monument to the erudition of its writer.

My pride had a fall, but, undaunted, and knowing, my colleagues, that you and I certainly must be scientific and therefore reputable professional men deserving of public honor, for does not our constitution say, after we have signed it (as it also provides, that we shall), that we are "Stated and Honorary Members," whose "purpose and objects" are "to contribute to the diffusion of true science and particularly the knowledge of Veterinary Medicine and Surgery.'' Do I not find upon our printed list of Honorary Members seven names? True, five are dead. Do I not find upon the Secretary's roll two hundred and seven names? It is again true that only about forty ever come to our meetings, and a vast number are so occupied with the diffusion of scientific knowledge and other duties that they have not time to obtain their certificates of membership and pay their dues. But, such a number of the most distinguished Doctors of the land, bound by such a code of ethics as we also find in the Constitution, must surely be ''wights of high renown,'' and I again fell to dreaming of our celebrity, of our usefulness not only as practitioners to the individual, but as health officers for the common weal, as economists for the great agricultural community in their dealings in motors and food animals, as experimental pathologists who verify the work of, and offer medical hypotheses to our own sister profession, Human Medicine.

I searched the lexicons for terms which would give us sufficient praise. The story of Solomon and the lily brought in an allusion to Botany, to be sure, but was scarcely strictly veterinary. The one about the Lion's skin as another's robe, certainly was a veterinary simile, and reminded me somewhat of certain discussions at Medical Society meetings, but still, was not quite appropriate; at last I thought of the tale of the turtle and the hare, and was so *well* satisfied that I put down my pen, folded up my tents, and stole away to other occupations for a week. During which time this eventful meeting in Brooklyn did not enter my head. To my surprise when I took up the subject again, I found that other people's hares were arriving everywhere and my turtle had not finished an address to you for me, nor indeed had he accomplished anything. I studied over the matter, convinced still that my turtle was a good simile, and I became more convinced of it the farther I went. Veterinary Medicine, like the turtle, must drag itself from a lowly origin. Its advance must be slow, through the mire of public prejudice, entangled by the reeds of quackery on all sides; when in clear water it must not resent attacks, but draw in for the moment, safe in its own solid usefulness, to come to the surface again and climb to the shore at the proper time. But, as I said, my turtle had done nothing and I was in despair.

Then I took to studying turtles. Terrapin looked the most attractive to begin with, but it has only one use. The great green turtle does not live in my neighborhood, and I could not investigate its qualities. However, the mud turtle abounds, and the more I became acquainted with this noble animal the closer I found the relationship between it and our Association to be. Gentlemen, the mud turtle has good qualities, and when induced to move is capable of going long distances, but it has one sad trait—it is *lazy*.

I then wondered if only I and the Committees I had appointed for the last year were lazy, or if it was an Association characteristic. I went back to the records and with your permission will replace an account of what we have *not* done during the last year by a summary of what has been done before us for a quarter of a century, and an outline of what we may do in the next.

*Formation United States Veterinary Medical Association.*—(First meeting, 1863). On the ninth of June, 1863, according to a previous arrangement, a large number of Veterinary Surgeons and Practitioners met at the Astor House, New York. Wm. A. Wisdom, of Delaware, stated the object of the meeting and read the minutes of two meetings which had been held in Philadelphia, the previous year. Dr. John Busteed, of New York, was appointed chairman to organize the meeting, and R. Jennings, of Bordentown, was appointed Secretary.

The following States were represented at this first meeting: New York: John Busteed, A. S. Copeman, R. H. Curtis, A. Liautard, A. Large, Louis Brandt, C. C. Price, W. Bannister, Wm. J. M. Cown, John Budd, E. Nostrand, C. Burden and James Mulligan. Massachusetts: Chas. M. Wood, E. F. Thayer, Wm. Saunders, R. Farley, J. H. Stickney, James Penniman, O. H. Flagg and R. Wood. Maine: E. F. Ripley. Pennsylvania: R. McClure, G. Mellor, J. C. Essenwein, E. H. Palmer and Isaiah Michener. Delaware: Wm. A. Wisdom. Ohio: G. W. Bowler. New Jersey: Jacob Dilts, J. C. Higgins, W. R. Mankin, R. Jennings, A. Philips, Jacob Philips, J. C. Walton, A. C. Budd and S. Humphrey. From London were, J. K. Quickfall and John Arnold.

They appointed the following Committee to draft Constitution and By-Laws, and the meeting adjourned until nine o'clock the following morning: J. H. Stickney, chairman; G. W. Bowler, A. S. Copeman, Isaiah Michener, Jacob Dilts, E. F. Ripley, Wm. A. Wisdom, Dr. Jno. Busteed, ex-officio.

On June 10, 1863, at 9 A. M., Dr. Busteed called the meeting to order, and Dr. J. H. Stickney, Chairman of the Committee, read a draft of a Constitution and By-Laws which were adopted.

An election for officers resulted in the choice of President, Dr. J. H. Stickney; Vice Presidents, R. H. Curtis, New York; Wm. Saunders, Massachusetts; E. F. Ripley, Maine; R. M. McClure, Pennsylvania; Wm. A. Wisdom, Delaware; G. W. Bowler, Ohio; R. Jennings, New Jersey. Recording Secretary, Dr. A. Liautard, New York. Corresponding Secretaries, Wm. J. McCown, New York; R. Wood, Massachusetts; Isaiah Michener, Pennsylvania; J. C. Walters, New Jersey. Treasurer, A. S. Copeman. Censors (6), A. Large, C. M. Wood, E. H. Palmer, E. F. Thayer, Jacob Dilts, J. C. Essenwein.

A resolution of thanks of the Association was adopted to Dr. J. Busteed for his "efforts and impartiality in organizing the meeting," and to R. Jennings and R. McClure for "their zeal and perseverence in forming the previous meeting in Philadelphia."

Dr. J. Busteed moved that the Association appoint an Orator for the ensuing Anniversary Meeting, but declined acting as such himself.

R. McClure gave an address on The Origin and Importance of Veterinary Education and Science.

R. Jennings presented some pathological specimens with an address, which was ordered to be placed on record, and Chas. M. Wood read an article on Veterinary Education, after which the meeting was adjourned until the first Tuesday of September, 1864.

*First Semi-Annual.*— On January 19th, 1864, there was a meeting of the Comitia Minoria held in New York. It was called to order by the President. Some difficulty seems to have existed over the status of some gentlemen as original members, they having been present at the Philadelphia meetings, but not at the organization in New York. R. Jennings presented a paper (title not given) and read one from G. W. Bowler on Rabies.

*First Annual.*— On September 6th, 1864, the annual meeting was called to order, at the Astor House, New York, by the President, J. H. Stickney.

Dr. Busteed, Library Committee, reported that he had not received any contributions for the Library.

R. Jennings presented the Association with one copy of "The Horse and His Diseases," one copy "Cattle and Their Diseases," and one on "Sheep, Swine and Poultry," for the Library, which seems to have been the only active measure ever taken in that direction.

The Corresponding Secretaries were reduced to one, and the Board of Censors increased from six to nine, which, however, appears never to have been fulfilled.

The following papers were presented: A. S. Copeman, "Composition and Fundamental Properties of the various Tissues of Animals." G. W. Bowler, "Atrophy of Kidneys in two Calves." R. Jennings, "Suppression of Urine in a Horse." C. M. Wood, "Status of Veterinary Science in the United States." A. S. Copeman, "Physical and Vital Forces."

An election of officers resulted in choice of President, A. S. Copeman; Vice Presidents, R. H. Curtis, Wm. Saunders, C. F. Ripley, G. Mellor, W. A. Wisdom, G. W. Bowler and J. C. Walton; Corresponding Secretaries, W. J. M. Cown, O. H. Flagg, E. Nostrand, S. Humphrey; Treasurer, C. M. Wood; Censors, C. C. Grice, New York; E. F. Thayer, J. H. Stickney, Massachusetts; A. Philips, New Jersey; Isaiah Michener, Pennsylvania; A. Liautard, New York.

The death of Edwin H. Palmer, of Pennsylvania, was announced.

Committees: Education, Liautard, Wood and Bowler; Finance, Saunders, E. H. Birney and Ripley; Library, J. Busteed, Jennings and Grice; Diseases, Stickney, Thayer and Michener.

*Second Semi-Annual.* — On March 7, 1865, the Comitia Minora held a meeting at the office of Dr. Liautard, in New York, at which there was no action of note.

*Second Annual.*—The second annual meeting was held at Young's Hotel, Boston, on Sept. 5, 1865. A. S. Copeman, President, in the chair. The Censors were empowered to fill vacancies in their board in order to obtain a quorum.

Henry Lawrence was admitted to membership. An election of officers resulted in President, C. M. Wood; Vice Presidents, W. J. M. Cown, W. Saunders, W. A. Wisdom, Isaiah Michener,

C. Higgins and E. F. Ripley ; Recording Secretary, C. Burden ; Censors, Messrs. Bowler, Stickney, Large, Liautard, Wood and Copeman.

A. S. Copeman, the retiring President, read an article on "Philosophy of the Sciences." A. M. McClure was expelled for ungentlemanly conduct at the last meeting.

*Third Semi-Annual.*—March 5, 1866, a special meeting of the Comitia Minora was held at the New York College of Veterinary Surgeons, President C. M. Wood in the chair, at which a motion made at the annual meeting to hold the semi-annual at Philadelphia, was rescinded.

March 6, 1866, the semi-annual meeting was called to order in the N. Y. College of Veterinary Surgeons, at which action was taken as to the duties of Secretary, the employment of secret medicines and the qualifications of membership.

*Third Annual.*—The third annual meeting was held in the New York College of Veterinary Surgeons, on Sept. 4th, 1866. A committee was appointed to investigate the charges brought against R. Jennings (while acting as Secretary) of tampering with the records, also of withholding certain papers belonging to the Association. Upon their report Mr. Jennings was expelled from the Association. A committee was appointed to prepare a code of ethics, which they presented later in the day and which was adopted. Dr. J. Busteed was then elected an honorary member. Papers were read by H. Lawrence and one by A. S. Copeman on "Diseases of the Chest," followed by a discussion on Navicular Disease.

The following officers were elected : President, R. H. Curtis ; Vice Presidents, E. F. Ripley, W. A. Wisdom, A. Philips, G. W. Bowler, J. F. Budd and O. H. Flagg ; Recording Secretary, C. Burden ; Treasurer, E. F. Thayer ; Corresponding Secretaries, Messrs. Cown, Michener, Walton and Stickney ; Censors; C. M. Wood, Stickney, Large, R. Wood, Saunders and Liautard.

*Fourth Semi-Annual.*—The fourth semi-annual meeting of the Comitia Minora was held at Young's Hotel, in Boston, March 5, 1867, R. H. Curtis, President, in the chair. The Comitia Minora reported the Constitution and By-Laws revised and with Amendments, and the Secretary was ordered to have printed one hundred copies.

*Fourth Annual Meeting.*—The fourth annual meeting was called to order in the N. Y. College of Veterinary Surgeons on Sept. 3, 1867. The minutes of the semi-annual meeting with revised Constitution were read and the latter adopted with one alteration, viz: that the time of holding the annual meeting be the third Tuesday in September.

An election for officers resulted in choice for President, Robert Wood; Vice President, W. H. Wisdom; Treasurer, E. F. Thayer; Recording Secretary, J. F. Budd; Corresponding Secretary, J. C. Walton; Censors, C. M. Wood, J. H. Stickney, A. Large, A. Liautard, W. Saunders, O. H. Flagg.

A. Large and A. Liautard reported disease existing on Long Island which they named "Cerebro Spinal Meningitis," and C. M. Wood one on Scrotal Hernia.

Drs. C. M. Wood, Stickney, Thayer and Large were appointed a committee "to review and revise the transactions of the past and future," and it was resolved that all the transactions of the Association up to the present time be published in one volume.

*Fifth Semi-Annual.*—The fifth semi-annual meeting was again held in New York at the same place, on March 5th, 1868. An addition to Article VIII. of the Constitution was offered providing that all members two years in arrears of their dues should have their names dropped from the rolls. A committee consisting of Messrs. Large, Liautard, Stickney, C. M. Wood, and Thayer were appointed to "investigate the subject of printing a Veterinary Journal." This meeting was honored by the presence of Prof. Gamgee of London.

*Fifth Annual Meeting.*—The fifth annual meeting was held at Young's Hotel, Boston, September 1st, 1868.

The action in regard to a special Committee, revising and publishing the transactions of the Association was reconsidered, and this duty was given to the Committee on Intelligence and Education. James L. Robertson, of New York, and I. S. Lombard, of Boston, were admitted as members. The resignation of R. H. Curtis was accepted and he was elected an honorary member. The officers of the last year were re-elected, President, R. Wood; Vice-President, W. A. Wisdom; Secretary, J. F. Budd; Treasurer, E. F. Thayer; Censors, C. M. Wood, A. Liautard, J. H. Stickney, A. Large, W. Saunders and O. H. Flagg.

A motion was carried:

That the United States Veterinary Medical Association as a body protest against the appointment by the general government through the recommendation of General Grant of Mr. Alexander Dunbar as a Clinical Lecturer to the Army Veterinary Surgeons and Farriers, for an alleged discovery of a mode of treatment of the disease of the horses feet, the operation being no discovery, but a regeneration of an obsolete idea and worthy of the attention and patronage of the Society for the Prevention of Cruelty to Animals. It being both an evidence of ignorance and barbarity, furthermore Mr. Dunbar has no claim whatever to the title of veterinary surgeon, either by education or professional association.

*Sixth Semi-Annual.*—The sixth semi-annual meeting was again held at Young's Hotel, Boston, on March 16th. 1869. The following important resolution was adopted :

*Whereas*, the United States Veterinary Medical Association, founded in June, 1863, has issued diplomas of membership to members of said Association, and whereas the issue is illegal, the Association not being incorporated or chartered, it is recommened by the *Comita Minora* to the Association that the issues of diplomas to new members be stopped ; that all the old diplomas be recalled by the Secretary, and that sixty days after said record the Secretary be ordered to publish a notice in the newspapers, notifying the public that all diplomas claimed or exhibited as coming from said Association are illegal, consequently of no professional value, and that instead of said "diploma" the Association only issue a private receipt of the initiatory fee.

The death of the former President, R. H. Curtis, was announced.

*Sixth Annual Meeting.*—The sixth annual meeting was held in New York, September 21st, 1869.

Resolutions of regret were passed at the loss sustained in the deaths of the former Presidents, R. H. Curtis and C. M. Wood.

It was resolved to substitute the word "members" in the constitution and by-laws, wherever the word "fellow" existed.

Dr. Walter Burnham, of Lowell, Mass., was elected an honorary member.

It was resolved to petition Congress for an act of incorporation of the U. S. Veterinary Medical Association.

The Committee on Intelligence and Education reported that they found no papers in the hands of the Secretary worthy of publication. The following officers were elected :—President, E. F. Thayer ; Vice-President, I. Michener ; Corresponding Secretary, O. H. Flagg ; Recording Secretary, J. L. Robertson ; Treasurer, C. Burden ; Censors, R. Wood, J. H. Stickney, A. Liautard, W. Saunders, J. C. Walton and A. Large.

An interesting discussion took place at this meeting on "spaying of cows," Dr. Busteed claiming that the operation was first introduced by an American named Thomas Quina. (?)

*Seventh Semi-Annual.*—On March, 15th, 1870, the seventh semi-annual meeting was called to order at Philadelphia, but no quorum was present.

*Seventh Annual Meeting.*—The seventh annual meeting was called at the College of Veterinary Surgeons in New York, on Sept. 20th, 1870. The officers of the last year were re-elected. No papers appear to have been presented.

*Eighth Semi-Annual.* — The eighth semi-annual meeting held at Young's Hotel, Boston, March 21st, 1871, was occupied only by discussion on the eligibility of candidates for membership.

*Eighth Annual Meéting.* — The eighth annual meeting, again at New York at the College of Veterinary Surgeons, on Sept. 19th, 1871, was called to order by Dr. Thayer.

The following resolution was adopted and a copy sent to the Board of Trustees of the New York College of Veterinary Surgeons:

That the U. S. V. M. Association request the Board of Trustees of the N. Y. C. V. S. to have an examination of students before admission to the course of lectures, of such a nature as may seem to them to further the object of a higher grade of education.

Fred. L. Thayer, M.D., was admitted as an honorary member. Peter Nostrand was admitted as a member. Drs. Stein, Weisse and Percy, members of the faculty of the N. Y. C. V. S., were admitted as honorary members, and Robert J. Saunders as a stated member. A ballot for officers elected :—President, A. Large ; Vice-President, W. Saunders ; Recording Secretary, J. L. Robertson ,Corresponding Seoretary, O. H. Flagg ; Treasurer, C. Burden. Censors, R. Wood, J. H. Stickney, A. Liautard, W. Saunders, J. C. Walters add E. F. Thayer.

Dr. Liataurd presented a paper on Cerebro Spinal Meningitis.

*Ninth Semi-Annual.*—The ninth semi-annual meeting held in Boston at Young's, March 16th, 1872, received a report from the Committee on Charter, stating that after legal advice they deemed it impracticable to obtain the same from the United States Government.

*Ninth Annual Meeting.*—The ninth annual meeting was called to order in New York at the College of Veterinary Surgeons, on September 17th, 1872. After routine business, Theo. K. Very and Robt J. Saunders were elected members. Officers for the next year were elected as follows:—President, A. Large; Vice-President, W. Saunders; Recording Secretary, J. L. Robertson; Corresponding Secretary, O. H. Flagg; Treasurer, C. Burden. Censors: Messrs. R. Wood, Stickney, W. Saunders, E. F. Thayer, Walton and Liautard.

Dr. Thayer presented a case of Fistula of the Parotid Duct and Cerebro Spinal Meningitis was discussed.

*Tenth Semi-Annual.*—The tenth semi-annual meeting was again held in Boston, March 17th, 1873, at which the death of Dr. Watson was reported.

*Tenth Annual Meeting.*—On Sept. 16th, 1873, the tenth annual meeting was held in New York.

J. D. Hopkins, R. W. Finley and Peter Peters were elected members. The officers of the past year were re-elected, except Messrs. Saunders and Walton, who were replaced by Messrs. Nostrand and Robertson, in the Board of Censors.

*Eleventh Semi-Annual.*—At the eleventh semi-annual meeting held in Boston on March 17th, 1874, it was proposed to donate fifty dollars in aid of the erection of a monument to the memory of Bourgelat at Alfort, France.

*Eleventh Annual Meeting*-—The eleventh annual meeting was not held owing to an error in the date of the notices which had been sent out.

*Twelfth Semi-Annual.*—The twelfth semi-annual meeting held in Boston, March 26th, 1875, seems to have been only a meeting of the *Comitia Minora*.

*Twelfth Annual Meeting.*—Sept. 21st, 1875, was held a the American Veterinary College, A. Large, the President in the Chair.

An appropriation was made for the monument of Claude Bourgelat.

A. Lockhart, New York, Chas. P. Lyman, C. H. Stocker, J. Gadsen, Chas. Michener, Philip Fernsler, John Meyer, Jr., C. T. Bell, E. Travers, John B. Cosgrove, Richard P. Blakeley were admitted as members.

At the election of officers, the following were chosen :—President, A. Liautard ; Vice-President, T. S. Very ; Corresponding Secretary, C. P. Lyman ; Recording Secretary, J. D. Hopkins ; Treasurer, C. Burden. Censors : R. Wood, Lockhart, Stickney, Flagg, Large, Robertson.

At this meeting it was ordered that two prizes be given each year to the best and second best paper on any Veterinary subject. That a prize committee be appointed to confer with the winners as to their choice of prize—either as to medal, books, or instruments to the amount of fifty dollars. The Secretary notified the members of the above resolution. The Prize Committee consisted of Messrs. Large, Stickney and Lyman.

*Thirteenth Semi-Annual Meeting.*—Was held at Young's Hotel in Boston, on March 21st, 1876.

Dr. Thayer presented a paper on Epizootic Aphtha and Dr. Lyman one on Internal Disinfection.

On April 28th, a Special meeting was held at the Haynes House, Springfield, Mass., at which an effort was made to propare a veterinary Exhibit at the International Exposition to be held in Philadelphia, which, however, appears to have been a failure.

*Thirteenth Annual Meeting.*–Was called to order at the American Veterinary College in New York, on September 10th, 1876.

Mr. Henri Bouley, General Inspector of Veterinary Schools in France, and George Fleming, F. R. C. V. S. Veterinary Surgeon, Royal Engineers, were elected honorary members.

The officers of the last year were re-elected.

This meeting was honored by the presence of Prof. McEachran, of Montreal, and Mr. Duncan, who represented Prof. Smith, of Toronto, Canada.

It was resolved that a Journal be printed by the Association, semi-annually, January and July 1st, to be called "The American Veterinary Review," A. Liautard and A. Lockhart Editors, to cost each member fifty cents per volume, balance of the expense to be paid from the Association funds.

The meeting adjourned to meet at the Continental Hotel, Philadelphia, September 20th.

The adjourned meeting was called at the Continental Hotel on the morning of the 20th. The President, Prof. Liautard in the chair.

The following papers were presented :—Prof. Liautard, "History of Veterinary Medicine in the United States." Prof. Law, "Zymotic Diseases, and the Duties of the Veterinary Surgeon." A. A. Holcombe, "The Effects of Stimulants in Disease." Prof. McEachran, "Sanitary Measures in Preventing Diseases in the United States and Canada." Thomas S. Very, "Chronic Lameness in Horses." E. F. Thayer, "Fistula."

The meeting was again called on the morning of September 21, and was occupied with discussions of the papers of the previous day.

*Fourteenth Semi-Annual.*—Returned to Boston, March 20th, 1877.

It was resolved that the *American Veterinary Review* should be published monthly and that the faculty of the American Veterinary College be added to the Editoral Staff.

It was resolved that all papers for the Annual prize shall be presented to the President of this Association before the fifteenth of July of each year. A committeee was appointed to petition Congress in the enactment of more stringent laws for the prevention of disease by the importation of cattle from foreign countries.

A number of cases of pathological specimens were presented which was followed by a paper by A. A. Holcombe on Spinal Meningitis.

*Fourteenth Annual Meeting.*—Was held at the American Veterinary College, September 18th, 1877.

Messrs. John Meyer, Sr., W. J. Coates, C. H. Hall, Geo. P. Peniman and C. H. Peabody were elected members.

C. P. Lyman was elected President ; William Bryden, Vice-President ; A. A. Holcombe, Recording Secretary ; Ernest Traver, Corresponding Secretary ; Chas. Burden, Treasurer. Censors : Messrs. Robertson, Lockhart, Stickney, Holcombe, Lyman and Liautard.

Dr. Liautard, the retiring President, in his address called special attention to the great step which had been taken in establishing a journal for the profession.

It was ordered that the editor of the *American Review* be allowed to spend an amount of money for advertising said *Review*, said amount to be left to his own judgment. An assessment of $5.00 per member was made to replace neglected dues and subscriptions to the *Review*.

*Fifteenth Semi-Annual.*--Met at Boston, March 19th, 1878.

Communications were received from the American Veterinary College, Cornell University, Illinois Industrial University, Montreal Veterinary College and the Toronto Veterinary College in regard to the proposed Congress of American Veterinary Colleges in which each seemed afraid that they would be called upon to meet the representatives of some College in disreputable standing, after which the Committee on Education and Intelligence were instructed to continue their efforts toward calling a convention of the teachers of the various Veterinary Colleges of America, for the purpose of improving the standard of Veterinary Science and the Curriculum of Studies in said Colleges.

Prof. Eachran was elected to honorary membership, and J. A. Brackin, J. C. Fries, J. C. Mallory and J. C. Fogg to be active members.

The Editor of the "American Veterinary Review" reported the condition, circulation, expense, etc., of said Journal, and asked for the privilege of increasing the size and reducing its subscription price from $5.00 to $4.00 which was granted.

Dr. Bryden presented a paper on "Spavin" and Dr. Liautard on "Parenchymatous Injections." A large number of cases were also reported.

*Fifteenth Annual Meeting.*—Was held at the American Veterinary College, on Tuesday, September 17th, 1878.

The following new members were elected :—John F. Winchester, W. L. Schmidt, A. H. Rose, S. S. Field, W. H. Wray, W. Murphy, J. MacLaughlin.

The following officers were elected :—President, C. P. Lyman ; Vice-President, W. Bryden ; Recording Secretary, A. A. Holcombe ; Corresponding Secretary, W. J. Coates ; Treasurer, C. Burden. Censors : Messrs. Robertson, Lockhart, Stickney, Holcombe, Thayer, Liautard.

A number of cases were presented. Dr. Liautard read an article on "Melanæmia." Dr. Liautard was unanimously reelected to the Editorship of the *American Veterinary Review*, and instructed to select such assistants as he chose, and to conduct the *Review* in whatever manner would in his judgment best conduce to its success.

Dr. Holcombe presented a paper on "Acute Inflammation of the Air Passages and Pulmonary Emphysema," arising from inhalation of vegetable smoke.

*Sixteenth Semi-Annual.*—Was held at Young's Hotel on the 18th of March 1879.

It was called to order by the Secretary in the absence of the President and Vice President. Dr. Liautard was elected Chairman, pro. tem.

It was ordered that after the next Annual Meeting this Association pay to the Secretary an Annual fee of $20.00.

*Sixteenth Annual Meeting.*—Was held at the American Veterinary College, September 16th, 1879.

Messrs. O. C. Frailey, R. M. McLean, W. B. E. Miller and T. J. Herr were elected members.

The following officers were elected: President, J. L. Robertson; Vice President, J. H. Stickney; Recording Secretary, A. A. Holcombe; Corresponding Secretary, W. J. Coates; Treasurer, C. Burden; Censors, Messrs. Liautard, Lyman, Lockhart, Thayer, Coates and Michener.

Dr. Hopkins presented a paper on "Contagious Pleuro-Pneumonia." Dr. Liautard presented one also on "Rupture of the Flexor Metatarsi."

The Committee on prizes reported that two papers had been presented, but that they had determined to give no prize to either author and recommended the return of the papers to their authors. Dr. Michener presented one on "Cerebro Spinal Meningitis."

A Committee was appointed to draw up a set of resolutions to be presented to Congress in relation to the investigation and prevention of contagious diseases of domestic animals. This Committee met on Tuesday, Nov. 9th, at the Hotel Royal, New York City, and again in December at the American Veterinary College, when the petition relating to animal diseases was completed, of which 600 copies were printed and sent to members of Congress, to the heads of Departments of the Government and to Members of the Association.

*Seventeenth Semi-Annual.*—Was held in Boston on March 16th, 1880.

F. S. Billings, W. J. O. Sullivan, Chas. Winslow, Wm. L. Zuill, Walter H. Hornblower, Edgar P. Wing, Herbert T. Foote, Thos. C. Cowhey and Geo. H. Bailey were elected members.

Dr. Peabody presented a paper on "Chloral Hydrate" as an anasthetic in operations.

*Seventeenth Annual.*—Was held at the American Veterinary College on September 1st, 1880.

H. B. Boyd, W. H. Gryce, A. P. Weeks, R. Hall, P. Z. Colsson and J. Gerth, Jr., were elected members.

J. L. Robertson was elected President; O. H. Flagg, Vice President; C. B. Michener, Secretary; C. Burden, Treasurer, and Messrs. Liautard, Lyman, Lockhart, Thayer, Coates and Michener, Censors.

Dr. Liautard presented a paper on "Osteo Sarcoma."

*Eighteenth Semi-Annual.*—Was held in Boston, March 13th, 1881. Drs. E. Hansherr, D. Cochran, and Thomas Lockwood were elected members. A communication from Dr. Holcombe in relation to Veterinary Medicine in the U. S. Army, was one of the foundation stones in the agitation of this subject in the country.

A Committee upon Army Legislation was appointed, consisting of Dr. Michener, L. McLean and Bryden.

Dr. Peabody presented an essay on "Phthisis Pulmonaris Verminalis.

Dr. Robert Wood read a paper on "Sarcocele."

The resignation of Prof. Liautard, as editor of the American Veterinary Review was accepted. The Association then tendered to Prof. Liautard, free of all encumbrance the American Veterinary Review as a slight recognition in respect to the labor he had done for us and the perfection of the paper. Dr. Liautard accepted the gift with the assurance that he would strive not only to keep the review up to its present standard, but would improve it whenever possible.

*Eighteenth Annual Meeting.*—Was held at the American Veterinary College, September 20th, 1881.

Drs. John Dougherty, R. H. Harrison, M. Bunker, J. E. McNichol, D. J. Dixon, W. W. Burth, Jos. Bushman, and F. H. Osgood were elected members.

At the election of officers: Wm. Bryden was the choice for President; L. McLean, Vice President; C. B. Michener, Secretary; C. Burden, Treasurer; Drs. Liautard, Lymans, Lockhart, Saunders, Robertson and Michener, Censors.

The annual dues were reduced to $2.00. The initiation remaiing as before, $5.00.

*Nineteenth Semi-Annual.*—Was again held in Boston, March March 21st, 1882.

Fully one-third of the members were present.

John Duane, Jr., and F. W. McClellan were elected members.

Dr Thayer presented a paper on "Osseous Nasal Polypus," which was followed by a general discussion on diseases of bone.

*Nineteenth Annual Meeting.*—Was held at the American Veterinary College, September 19th, 1882.

Drs. Fred. Saunders, Chas. L. Moulton, F. Traver, S. Kent, Jr., L. H. Howard, H. W. Atwood, W. C. Deboe, W. Dougherty, J. L. Leighton and W. A. Sherman were elected members. The officers of the past year were re-elected.

Dr. Liautard presented a paper upon inocculation for Anthrax. Dr. Stickney and Miller presented cases.

*Twentieth Semi-Annual.*—Was held in Boston, March 20th, 1883, Drs. F. H. Frinck, J. Hawkins, Andrew Sharp, A. F. Newton, L. M. Crane were elected members.

The Comitia Minora were delegated power to appoint a Representative to the International Veterinary Congress to be held at Brussells.

A number of cases and specimens were presented by Drs. Bryden, Stickney, Michener and Howard.

*Twentieth Annual Meeting.* — Was held at the American Veterinary College, September 18th, 1883.

Drs. Huntington, W. H. Hoskins, R. Kay, W. C. Brotherton, W. D. Atritcherson, Austin Peters, Cotton, E. A. McLane, B. D. Pierce, F. E. Rice, J. Skally, C. T. Goentner, Alex. Glass, J. C. Gardner, W. H. Pendry and F. J. Hansherr were elected members.

The election for officers resulted in the choice of W. B. E. Miller, President; W. J. Coates, Vice President; C. Burden, Treasurer; C. B. Michener, Secretary; Drs. Liautard, L. McLáne, Robertson, Hoskins, Lockhart and Stickney, Board of Censors.

*Twenty-first Semi-Annual.*—Was called to order at Young's Hotel, Boston, March 18th, 1884.

Drs. E. Burket, B. L. James, and R. S. Huidekoper were elected members.

*Twenty-first Annual Meeting.*—For the first time was held west of the Alleghenies, at the Grand Hotel, Cincinnati, Ohio, September 16th, 1884.

Drs. W. R. Howe, J. H. Detmers, and D. M. Schaeffer were admitted as members.

The Board of Censors was increased to seven. The death of William Saunders was announced.

The election of officers resulted as follows: President, W, B. E. Miller; Vice President, L. H. Howard; Secretary, C. B. Michener; Treasurer, C. Burden; Censors, Drs. Liautard, Robertson, Hoskins, J. C. Meyer, Geo. Corliss, Bryden and Crowley.

A Committee of three was appointed to confer with the Faculty of the Veterinary Colleges and Schools of North America to discuss the advisability of adopting an equal standard of excellence on examination. The committee consisted of Drs. Hoskins, Howe and Bryden. A number of papers were presented.

*Twenty-second Semi-Annual.*—Was held at Young's Hotel, March 17th, 1885.

Drs. Walton, Agersborg, Hawk, W. H. Leland, Dyer and Humphrey were admitted as members.

*Twenty-second Annual Meeting.*—Was held at the American Veterinary College, December 15th, 1885. The following officers were elected for the year:

President, L. McLane; Vice President, J. B. Cosgrove; Treasurer, J. L. Robertson; Secretary, C. B. Michener; Censors, Drs. Dixon, Lockhart, Corliss, Crowley, Miller, Field and Osgood.

Action was again taken in regard to the position of Veterinary Surgeons employed in the Army.

Dr. Liautard offered to add a gold medal of the value of $50.00 to the prize already offered by the Association for the best paper presented, which was accepted.

*Twenty-third Semi-Annual.* — Was held in Boston. Owing, as proper notice had not been given there was no session of the Comitia Minora. The legality of the meeting was questioned and the remainder of the day was occupied by the discussion of a few cases and papers.

*Twenty-third Annual Meeting.*—Was held at the Rossmore Hotel, New York, September 21st, 1886.

Seventeen new members were admitted: Drs. D. E. Salmon,

Francis Bridge, Jas. Walrath, R. C. Jones, C. S. Breed, D. D. Lee, K. Winslow, E. C. Beckett, Thos. Bland, G. C. Vanwater, W. S. Cuff, Wm. Rose, T. S. Butler and Wm. R. Harris.

Dr. Liautard was chosen President for the ensuing year. Drs. Zuill, Vice President; C. B. Michener, Secretary; James L. Robertson, Treasurer; Messrs. Lyman, Huidekoper, Dixon, Field, Rose, L. McLane and Osgood, Censors.

$100.00 was appropriated towards the erection of a monument to Henri Bouley at Alfort, France.

A prize was awarded to a paper to P. S. Butler.

*Twenty-fourth Semi-Annual.*—Was held at the University of Pennsylvania, Veterinary Department in Philadelphia, fifty members answered to the roll call. Fifteen new members were added to the roll.

After extensive Committee reports, Dr. D. E. Salmon presented a paper on "Contagious Pleuro Pneumonia," which was followed by an extensive discussion and resolutions in regard to the duty of the National Government to its suppression and prevention.

This meeting was honored by the presence of Professors McEachran and Lyford.

*Twenty-fourth Annual Meeting.*—Was held at the American Veterinary College, September 20th, 1887. Again fifty members answered to the roll call. Twelve new members were admitted. The following officers were elected for the year: President, Dr. Huidekoper; Vice President, J. C. Meyers, Jr.; Secretary, C. B. Michener; Treasurer, J. L. Robertson; Censors, Drs. Dixon, Lyman, Hoskins, Zuill, Rose, Burden and L. McLane.

*Twenty-fifth Semi-Annual.*—Was held at the rooms of the Medico-Chirurgical Faculty, Baltimore, Md., on March 20th, 1888. Again nearly half a hundred members answered to the roll call. Twenty-three members were admitted.

A resolution was adopted expressing confidence in the work of the Bureau of Animal Industry, and protesting against the passage by Congress of the Palmer Bill. Dr. Salmon presented a paper on "Hog Cholera" and another on the "Mediate Contagion of Contagious Pleuro-pneumonia." Dr. Clement presented a paper on the "Pathology of Contagious Pleuro-pneumonia" which was richly illustrated by a large collection of specimens, fresh and preserved, of the disease.

*Twenty-fifth Annual Meeting.*—Was held at the Rossmore Hotel, September 18th, 1888. Thirteen new members were admitted.

The following officers were chosen for the ensuing year: President, Dr. Huidekoper; Vice President, Dr. Dixon; Secretary, Dr. Hoskins; Treasurer, Dr. Robertson; Censors, Drs. Zuill, Rose, Winchester, Wray, Howard, Clements and McLane.

Dr. Huidekoper presented a paper on the "Origin of the Domestication of the Horse." After a discussion on Bovine Tuberculosis a committee was ordered to draft resolutions in regard to the contagiousness of this disease to man, and to present them to the Medical Congress then in session at Washington. Dr. J. C. Meyers, Sr., gave an account of the disease known as "Mad Itch in Cattle."

This completed the work of a quarter century. The meetings were always pleasant affairs socially, and whatever might have been the devergence of opinions during the day, and they have often been so great as to become personal, the evening reunited all present, and we learned to know each other and to fill the want of professional friendship which is felt by many who stand alone in new localities. Some meetings were replete with papers and it is to be regretted that we have not a record of many of the transactions. Other meetings, and they are unfortunately many of them, have been devoid of any public interest, either from lack of proper sense of duty on the part of those who might have furnished intellectual food to the others, or from, in many cases, the heedless interference, and delay of the proceedings by subjects of business (?) and trivial matters, which might have been left to one side. For the future we should remedy these errors. We should confine our "business" to the limits of what is absolutely necessary. We should take more accurate notes of cases and prepare papers and present them here, ready to defend their value. Veterinary literature has become an established fact in this country, and we should all aid in its improvement. The improvement of the education of our successors is a serious duty we have to perform. This lies not only with those connected with Veterinary Schools, but also with the whole profession, for it is the latter, who have the power to build up or tear down an institution by furnishing to it or withholding from it, students.

If they demand but little instruction, but a pittance will be given. If they will only study where every facility is given, the schools will vie with each other in increasing the facilities for a complete education. The improvement of the position of our colleagues in the Army offers a subject of national importance in which we must all aid. When we can see the Veterinarian of the Army an authority on the subject of Animal Industry, a factor of the government, a social peer of the best educated officers of the country, our profession throughout the land will have reached its proper recognition. We have a great deal to accomplish, but it can be done if we work together and are industrious.

